# A Rare Case of a Compressive Intracranial Epidermoid Cyst in a 13-Year-Old Patient

**DOI:** 10.7759/cureus.73197

**Published:** 2024-11-07

**Authors:** Waad Y Aldoseri, Raafat Hammad Seroor Jadah

**Affiliations:** 1 Medicine, Bahrain Defence Force Hospital, Riffa, BHR; 2 Pediatric Neurology, Bahrain Defence Force Hospital, Riffa, BHR

**Keywords:** epidermoid cyst, intracranial tumours, pediatric brain mri, pediatric neurology, pediatrics, neurology

## Abstract

Pediatric intracranial epidermoid tumors are rare, slow-growing benign cystic lesions primarily originating from the ectodermal cell line during human embryogenesis. Intraparenchymal epidermoid tumors typically present with headaches, seizures, and focal neurological deficits. Although CT of the brain may show non-specific findings, MRI studies are more reliable and have a high confidence value in diagnosing intracranial epidermoid cystic lesions. We report a young girl who presented with headaches and blurring of vision for a three-week duration. An MRI of her brain revealed a cystic lesion on the right side of the interpeduncular cistern and cerebellopontine angle (CPA) extending to the right optic chiasm, suggestive of an epidermoid tumor. The aim of reporting this case is to highlight the significance of having a high clinical suspicion of intracranial tumors based on the patient’s clinical manifestations.

## Introduction

Intracranial epidermoid cysts are rare congenital extra-axial tumors recognized as benign and slowly developing, accounting for ~1-2% of all intracranial tumors [1]. They are commonly located in the cerebellopontine angle (CPA, 40% of all cases), the fourth ventricle (5-18%), subarachnoid spaces of basal cisterns, and parasellar or sellar regions (~30%) [2,3]. Despite their sluggish growth, these tumors have a great tendency to adhere to vital neurovascular structures, causing impingement. The mass effect caused by impingement results in wide-ranging clinical presentations such as seizures, headaches, and diplopia [3]. Here, we report a rare pediatric intracranial epidermoid cyst presenting as transient bilateral blurring of the vision.

## Case presentation

A 13-year-old previously asymptomatic female with no previous significant medical or surgical history or known allergies presented to the ophthalmology outpatient clinic complaining of transient bilateral blurring of the vision. This symptom has been present for the past three weeks and has progressively worsened over time. The episode occurs in the morning when she awakens from her sleep and typically lasts for about ten minutes until her vision returns to normal. These episodes were linked to a brief history of nausea and dizziness, but there was no documented history of vomiting, loss of consciousness, abnormal movements, or seizures. However, there was a history of on-off headaches that happened throughout the day for the past three years; the headaches were throbbing in nature, mainly in the frontal area. The headaches occurred spontaneously without apparent triggers and were relieved by simple analgesics (paracetamol). Her medical and surgical history were unremarkable; she had normal developmental milestones and is up-to-date with all her vaccinations. On physical examination, the child was alert and cooperative, her vital signs were within the normal range, her blood pressure was 120/81 mmHg, and her growth parameters were on the 50th percentile. Her motor examination showed normal tone and power, with deep tendon reflexes (DTR) +2. Cranial nerve examination was normal, including examination of the fundus. There were no cerebellar signs and no signs of gait abnormalities. The rest of the systemic examinations were unremarkable.

**Figure 1:**
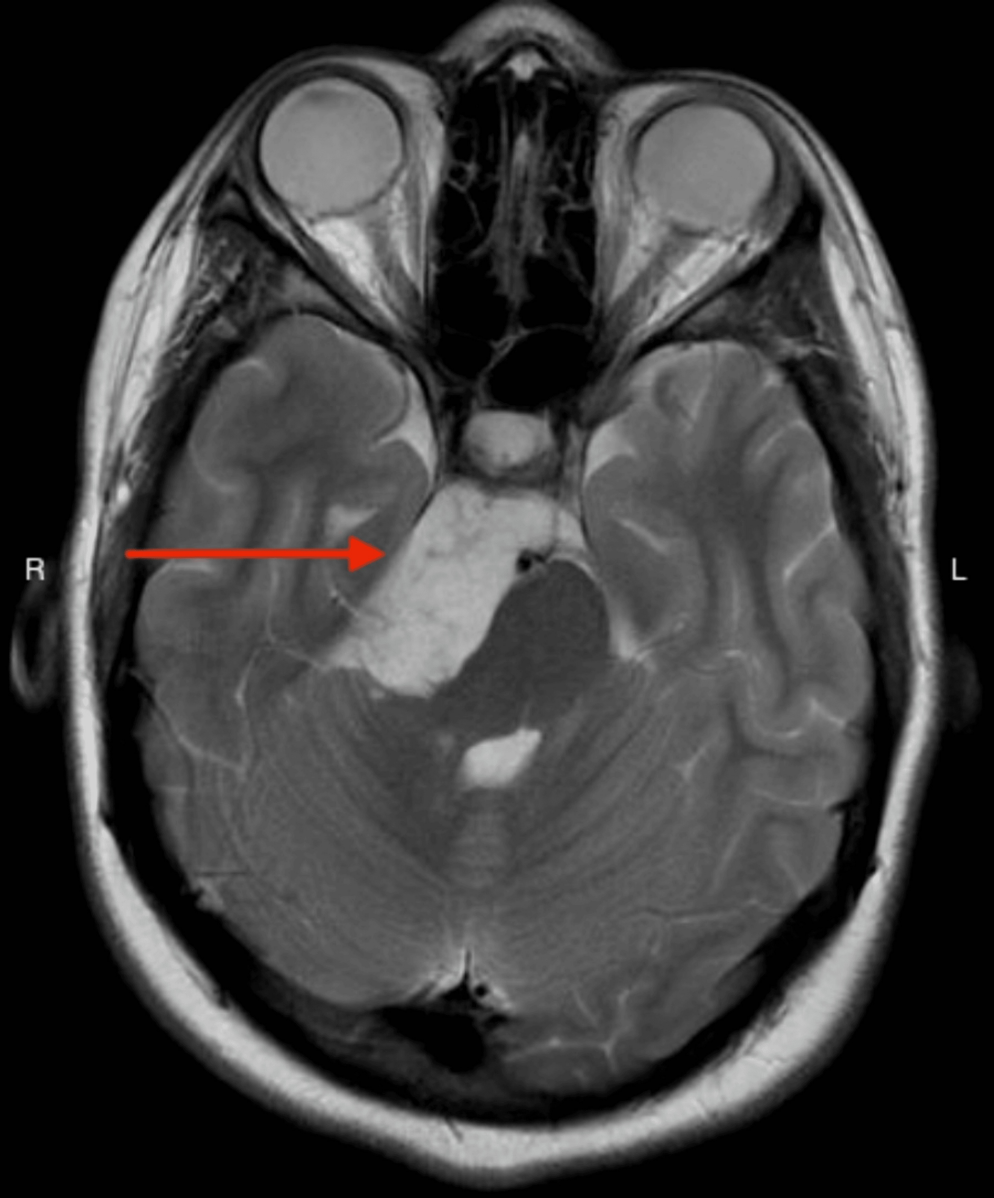
MRI of the brain Axial T2-weighted MRI image showing hyperintense space-occupying lesion (arrow). MRI: magnetic resonance imaging.

**Figure 2:**
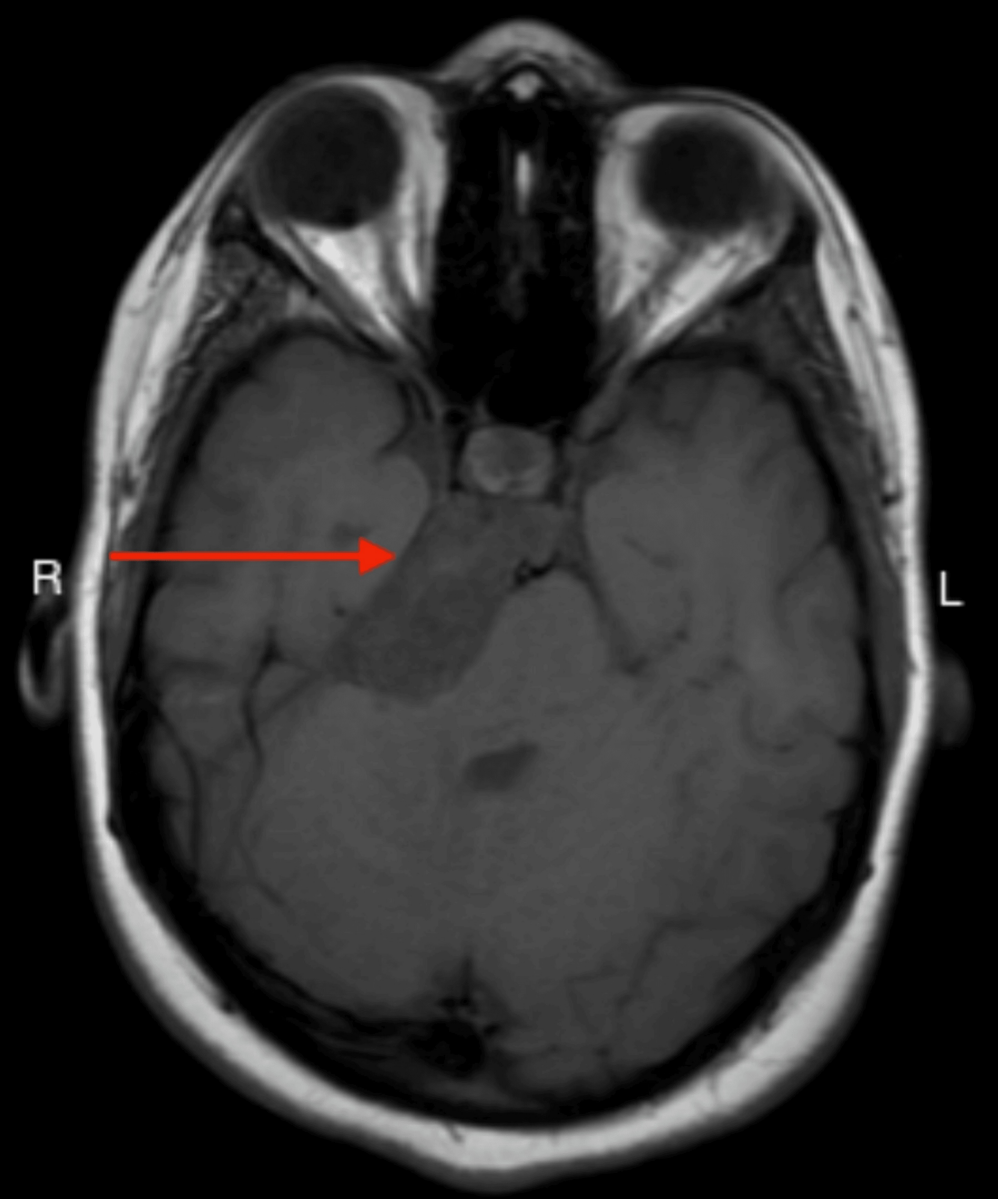
MRI of the brain Axial T1-weighted MRI image showing hypointense space-occupying lesion (arrow). MRI: magnetic resonance imaging.

**Figure 3:**
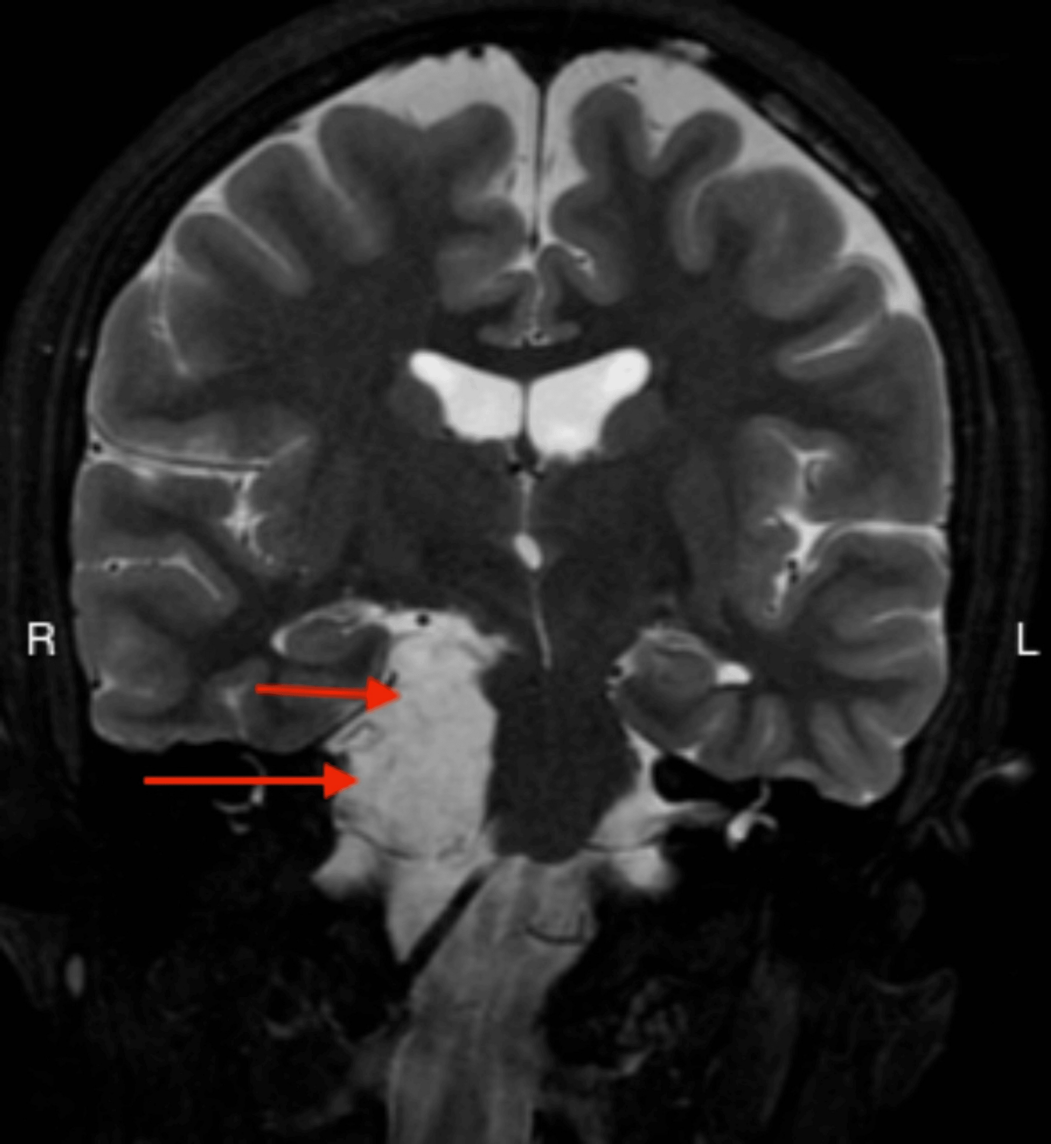
MRI of the brain Coronal T2-weighted MRI image showing hyperintense space-occupying lesion (arrows). MRI: magnetic resonance imaging.

Based on the patient’s clinical history, an MRI of the brain was performed, which revealed an extra-axial right-sided space-occupying lesion measuring 3.5 cm × 3.6 cm × 1.9 cm in the interpeduncular cistern, prepontine cistern, and cerebellopontine angle. The lesion extends to the posterior aspect of the suprasellar cistern in contact with the optic chiasm. The lesion has a significant mass effect on the pons and mesencephalon. It appears hyperintense on T2 and hypointense on T1, showing diffusion restriction with no enhancement, indicative of an epidermoid cyst (Figures 1-3). The neurosurgery team referred the patient for a consultation, advised a watch-and-wait approach, and did not recommend surgical intervention due to the benign nature of the tumor. The harm outweighs the benefit of surgery.

## Discussion

Intracranial epidermoid cysts represent 1-2% of all intracranial tumors [1]. They are rare congenital brain lesions originating from the neuroectoderm [4]. The basal cisterns' parasellar areas, the CPA, and the subarachnoid spaces are the most typical locations. The brain stem, spinal canal, cranial diploe, middle cranial fossa, and supratentorial intraparenchymal region are fewer common locations for epidermoid cysts [4]. These cysts are thought to form during early embryonic development, particularly between the third and fifth weeks of gestation, due to the displacement of dorsal ectodermal cells typically found in the midline [5]. Chandler et al. suggested that remnants of ectodermal tissue could persist on the inner or outer surfaces of the neural tube. This theory aligns with the development of epidermoid tumors within the brain's ventricles, parenchyma, or on its surface [6].

The epidermoid cyst location is determined by the sequestration time. A study by Fox et al. suggested that intra-parenchymal epidermoid cysts result from ectodermal elements sequestered within the neural tube during the third week of embryogenesis, when the primary central vesicle emerges. Conversely, the epidermoid would form in the CPA, middle ear, or orbital regions if sequestration takes place afterward (during the maturation of the secondary optic or cerebral vesicles). Intracranial epidermoid cysts sluggishly grow by cholesterol, keratin desquamation, and cellular debris accumulation, generating symptoms due to compression of nearby neurovascular structures [4].

Most intracranial epidermoid tumors present with neurological symptoms such as seizures, weakness, and headaches [7]. A study by Goel et al. evaluated clinical presentations of tentorium-based epidermoid tumors (located in the posterior and middle cranial fossa) in patients aged 7-62; results revealed the most common presentations to be headaches (55%), facial numbness (31%), vertigo (22%), ataxia (44%), hearing deterioration (18%), diplopia (14%), trigeminal neuralgia (13%), hemifacial spasm (10%), visual decline (9%), seizures (8%), vomiting (7%), and memory loss (4%) [8]. According to the study, most symptoms were secondary to the compressive mass effect on adjacent structures in the brain.

Another study by Kato et al. studied clinical presentations of intracranial epidermoid tumors in adult populations according to the tumor's location. Symptoms associated with epidermoid tumors in the CPA and ventricular system were hearing loss, trigeminal neuralgia, headaches, ataxia, hemifacial spasms, and diplopia [9]. In our case report, the patient is from a pediatric population; hence, few studies revealed the typical presentations of epidermoid tumors. No published studies reported transient blurring of the vision as the first clinical presentation in a pediatric patient, making the diagnosis challenging.

Brain MRI is a favorable neuroradiological investigation for intracranial epidermoid tumors. They characteristically appear as hyperintense lesions on T2-weighted images and hypointense on T1-weighted images due to the presence of keratin and cholesterol in the epidermoid, in addition to debris particles and protein that are responsible for the intensity of the homogenous and non-homogenous configurations [2], as appreciated in our report. MRI studies aid in diagnosing epidermoid tumors as they exhibit a precise diffusion restriction and bright homogenous signal. The treatment of choice is radical surgical excision of the tumor. The capsule of the cyst in intraparenchymal epidermoid tumors can be dense and adhere to the parenchyma surrounding them. This adherence can make dissection and total resection difficult in vulnerable regions. Furthermore, the true impact of recurrence on surgical results is uncertain, considering factors like the location of the disease, the degree of neurovascular involvement, and the extension pattern, which also affect resectability [10]. Consequently, the objective shifts to safe decompression with minimal risk of neurologic morbidities, such as in our case [2]. Presently, there are no targeted therapies or chemotherapeutic options for this type of tumor [11]. Despite their benign nature, epidermoid cysts can occasionally transform malignantly, particularly into squamous cell carcinoma, with a poor prognosis for most cases [2].

The primary indications for surgical resection of intracranial epidermoid cysts comprise cases in which the cyst is symptomatic, such as causing severe headaches, seizures, cranial nerve dysfunction, or additional neurological deficits. If the size or location of the cyst adds pressure on important brain structures or cranial nerves, surgery becomes necessary to relieve the compression. Surgery is also considered when the cyst obstructs cerebrospinal fluid (CSF) flow, causing hydrocephalus, or when the cyst has ruptured, which can lead to a chemical meningitis reaction. Although rare, surgical resection is warranted in cases of malignant transformation to squamous cell carcinoma [12].

The neurosurgical approach for resecting an intracranial epidermoid tumor depends on the tumor's size, location, and growth pattern. A study by Velho et al. outlines different surgical approaches according to the site of intracranial epidermoid tumors. Tumors located in the CPA are typically resected using a posterior retro-sigmoid approach with the patient in a lateral semi-sitting position for best access with minimal complications. Anterior interhemispheric surgical approach for interhemispheric tumors, pterional subtemporal approach for middle cranial fossa tumors, far lateral approach for infratentorial cisternal tumors, subfrontal approach for suprasellar or parasellar tumors, supracerebellar infratentorial approach for pineal region tumors, transcortical approach for interventricular tumors, and combined retro-sigmoid with subtemporal approach for bicompartmental tumors. Gross total resection is the treatment of choice, preserving adjacent neuronal and vascular compartments. Complications such as chemical meningitis can be reduced by administration of steroids preoperatively and avoiding spillage intraoperatively [13].

Complete removal of intracranial epidermoid cysts can be challenging due to their adherence to critical brain tissues, and often, only subtotal resection is feasible to avoid neurological damage. Continuous monitoring post-surgery is essential to detect possible recurrences.

## Conclusions

Pediatric intracranial epidermoid cysts are rare, slow-growing lesions that can present with significant neurological symptoms, and red flag symptoms warrant further investigations, such as early morning headaches, transient blurry vision, diplopia, early morning nausea, vomiting, and headaches waking up from sleep. However, in some cases, such as ours, it is crucial to have a high clinical suspicion in children presenting with uncommon symptoms, such as transient blurring of the vision, to achieve the best possible clinical outcome. While complete surgical resection is ideal for preventing a recurrence, it is often unfavorable due to the risk of damaging critical brain structures. Given the potential for recurrence and the rare possibility of malignant transformation, close monitoring with regular follow-ups is crucial for optimal management and long-term outcomes.
